# Platelets function assessment in patients qualified for cardiac surgery – clinical problems and a newer diagnostic possibilities

**DOI:** 10.1186/s13019-018-0820-8

**Published:** 2018-12-22

**Authors:** Kinga Kosiorowska, Marceli Lukaszewski, Jacek Jakubaszko, Katarzyna Kościelska-Kasprzak, Grzegorz Bielicki, Waldemar Gozdzik, Marek Jasinski

**Affiliations:** 1 Department of Cardiac Surgery, Wroclaw Medical University, Wroclaw, Poland; 2 Department of Anaesthesiology and Intensive Therapy, Wroclaw Medical University, Borowska 213, 50-556 Wroclaw, Poland; 3 Department of Nephrology and Transplantation Medicine, Wroclaw Medical University, Wroclaw, Poland

**Keywords:** Platelet receptors, Platelet dysfunction, Aggregometry, Multiplate

## Abstract

**Background:**

As the incidence of cardiovascular diseases increases, the use of antiplatelet therapy is widely recognized. This presents clinicians with the challenge of balancing the risk of thrombotic and bleeding complications. Platelet dysfunction is one of the causes of postoperative bleedings and their etiology is not fully understood. Platelets receptors *point-of-care* investigation is of a remarkable value in assessing patients risk of bleeding. Reliable assessment of platelet function can improve treatment. The aim of this study was to evaluate the activity of platelet receptors in patients qualified for cardiac surgery, taking into account organ dysfunctions and pharmacological therapy applied in these patients.

**Methods:**

Seventy-one cardiac surgical patients were analyzed before surgery using multiple electrode aggregometry with the use of the ADP test and ASPI test. The cut-off values were determined based on the manufacturer’s recommendations. Patients were divided into four groups: Group I (33/71 patients, without platelet dysfunctions), Group II (6/71 patients, ADP < 710 AU x min), Group III (13/71 patients, ASPI < 570 AU x min) and Group IV (19 / 71 patients, ADP < 710 AU x min and ASPI < 570 AU x min). Biochemical data defining the efficiency of the liver and kidneys, the list of preoperative drugs used and the requirement for transfusion throughout the study group were collected.

**Results:**

The study group included 41 males (57.7%) and 30 females (42.3%), mean age 66 years. The majority of patients (94.4%) had platelet counts within the normal range, but platelet function was impaired in more than half of the studied patients (53.5%). No relationship was found between the biochemical markers of the kidneys and liver and the function of the ADP and ASPI receptors, while receptors activities were related (rs = 0.72, *p* < 0.001), and both associated with platelet count (rs = 0.55, *p* < 0.001 and rs = 0.42, *p* < 0.001, respectively). Platelet receptors activity was not related to the postoperative need for any type of transfusion as well as the applied preoperative pharmacological therapy.

**Conclusions:**

Early identification of patients at high risk of bleeding, using *point-of-care* platelet function assessment tests, enables a targeted therapeutic pathway. Due to the variety of factors affecting the activity of platelets, finding a specific cause of this pathology is extremely difficult. According to our study, the correlation between platelet receptor disorders and mild to moderate liver and kidney injury has not been demonstrated. However, platelet receptors dysfunction has been shown to be associated with a decreased number of platelets.

## Introduction

The hemostatic system provides a natural balance between the coagulation and fibrinolysis, enabling normal and undisturbed flow in the blood vessels. Its main components are platelets, plasma proteins (factors and inhibitors of the coagulation system and fibrinolysis) and the blood vessel wall [1]. Platelets main role is to maintain normal hemostasis. They also play an important role in inflammation, immune processes, and cancer progression [2]. Although very dynamic, circulating platelets remain inactive and get activated only when a vascular injury occurs and then they protect the vascular system against uncontrolled blood loss and begin to form the hemostatic plug. The peripheral and close proximity to the vessel wall positioning of thrombocytes opposes shear forces aimed at separating the plug from the vessel wall through the active bloodstream [3]. There are many causes of the platelet plug formation disorders that lead to pathological bleeding. Excessive bleeding remains a serious complication in a cardiac surgery patients, which requires transfusions of blood products, and also involves the need of re-exploration for bleeding, which significantly increases the adverse outcomes and elevates the morbidity and mortality [4]. These bleedings may have a different etiology, both surgical and resulting from disorders in the coagulation process itself. Coagulation disorders occur most often in the form of platelet diathesis due to platelet deficiency or their dysfunction. Thrombocytopenia poses a serious therapeutic problem. Its etiology is of autoimmune or idiopathic nature, a symptom of systemic diseases, such as the result of acute infection, heparin-induced thrombocytopenia (HIT), liver disease, hemolytic-uremic syndrome (HUS), disseminated intravascular coagulation (DIC), but also decreased platelet production induced by bone marrow dysplasia, increased degradation and accumulation in the spleen [5, 6]. The majority of urgent patients qualified for cardiac surgery take antiplatelet drugs, that cause completely and irreversibly blocked platelet function. The standard laboratory tests of the coagulation system (PLT, INR, APTT) performed before each procedure have a small predictive value. Only thrombocytopenia of < 50 × 10^9^/ L is associated with significant hemostatic disorders [7]. Despite the fact, that the platelet count does not provide any information about their activation potential, it still remains to be the standard in the preoperative bleeding risk assessment in cardiac surgery patients. The activity of platelet receptors and the degree of their blockade are important parameters that should also be assessed in the preoperative setting. Long waiting time for the result of the standard laboratory tests pose also a serious problem especially in the situation of acute hemostatic disruption. The latest European recommendations regarding the management of massive bleeding and coagulopathy include alternative diagnostic methods based mainly on the *point-of-care* principle (POC), which provides a quick and a bedside assessment of coagulation disorders [8].

Vascular hemostasis is followed by platelet hemostasis (activation, adhesion, and aggregation of platelets), which is the prelude to the cascade of enzymatic processes with the final effect of clot formation [9]. A favorable platelet response to vascular injury and blood loss initiates a plasma coagulation cascade. On the other hand, this physiological defense reaction in atherosclerosis patients may cause thrombotic disease, which may result in a myocardial infarction or ischemic stroke [10]. The monitoring of platelet function is used in the diagnosis of acquired and congenital thrombocytopathy as well as in the optimization in the dosage of antiplatelet drugs, preoperative screening of patients qualified for surgery and to determine the possible need for blood products. Multiplate® Analyzer (Roche, F. Hoffmann-La Roche Ltd., Switzerland) is a *point-of-care* (POC) device that allows, in a very short time, the bedside assessment of the platelet activity. In patients undergoing cardiac surgery, coagulation diagnosis methods, such as thromboelastometry and the Multiplate® Analyzer, are finding a wider and wider application, entering the standards of the safe patient delivery through the procedure. The POC method for assessing the platelet responses to ADP was included in the Society of Thoracic Surgeons and Society of Cardiovascular Anaesthesiologists Guidelines for Blood Conservation Clinical Practice [11]. It allows the identification of patients who are insensitive to P_2_Y_12_ inhibitors, in particular to clopidogrel. It has a special application in patients qualified for urgent procedures because it allows skipping the recommended waiting period after discontinuation of antiplatelet therapy, in order to carry out the procedure safely and early enough (Class IIb, Level of Evidence C).

Diseases of the liver and kidneys are frequent acquired platelet dysfunctions. Thrombocytopenia is the most common accompanying problem affecting up to 55% of patients with end-stage renal insufficiency (ESRD) and up to 76% of patients with hepatic insufficiency [12]. Platelets also play an important role in the inflammatory response, which is the main process occurring in both liver fibrosis and kidney diseases [11]. Chronic kidney disease is associated with a high risk of cardiovascular disease. ESRD, due to the effect of uremic toxins, predisposes to both bleeding and thrombosis [11]. Renal replacement therapy slightly improves platelet function but does not completely eliminate the bleeding tendency [13]. The pathophysiology of bleeding is not fully understood, but it is not due to thrombocytopenia. Platelets in uremia present a defects in GPIb-V-IX-vWF complex, platelet content secretion and platelet GPIIb-IIIa receptor [11]. As renal failure progresses, the pro-thrombotic potential increases, which elevates the risk of mortality due to cardiovascular diseases. In patients with mild to moderate renal impairment, there is a tendency for adhesion and aggregation of platelets through the increased platelet activity as well as a weak response to antiplatelet therapy [14–16]. The liver, by the production of coagulation and anticoagulation factors, plays also an important role in the coagulation process. That is why its dysfunction is associated with various disorders. The platelets undergo qualitative and quantitative dysfunction. Vitamin K deficiency, production of aberrant coagulation cascade factors as well as hyperfibrinolysis and DIC occurs [17]. The vWF factor is the only coagulation factor not synthesized in hepatocytes, but in endothelial, megakaryocytes and subendothelial connective tissue and in liver failure its value is increased, which may result from increased endothelial production, limited hepatic metabolism, or compensation mechanism that support primary haemostasis [18, 19]. Platelet disorders are most often associated with thrombocytopenia in patients with chronic advanced liver disease. The reason for this is splenomegaly resulting from portal hypertension in patients with cirrhosis [20]. Up to 90% of the pool of correct platelets can be found in the enlarged spleen. Routine tests such as PT, INR and APTT are often prolonged in these patients. Acute and chronic liver failure also causes immunologic destruction of platelets [21]. Although there are many works linking kidney and liver diseases with dysfunction of the coagulation process at various levels, including platelet dysfunction, until now the precise etiology of coagulation disorders has not been fully understood.

A number of pharmacological agents used may also interfere with the platelet function. In addition to the targeted ASPI receptor inhibitors (aspirin), ADP (clopidogrel and prasurgel) and αIIβb3 integrilin blockers, also known as GPIIb-IIIa (abciximab, eptifibatide, tirofiban), which are well-known antiplatelet agents, other widely used: NSAIDs, antibiotics, statins may impair the function of platelets and thus contribute to the creation or deepening of the already existing platelet dysfunction [22]. This is particularly important in patients with platelet dysfunctions caused by internal disorders, in which they remain compensated, but only until their function is not additionally pharmacologically disturbed [23]. Among the cardiovascular drugs, nitrates and vasodilators are conducive to lowering the reactivity of platelets. Statins contribute to a decrease in the platelet activity and ability to aggregate [22]. The most important factors in determining the physiology and pathology of platelets are their quantity, quality and time of survival in blood [24]. Platelet count is a routine clinical study, but it is not a reliable predictor of bleeding risk and does not allow to determine the cause of thrombocytopenia. POC diagnostics, allowing quick assessment of platelet receptors function, seems to be an indispensable tool, complementing the clinical status of the patient against high-risk cardiac surgery procedures. As previously described, the platelet dysfunction, in addition to the drugs used by the patient, is influenced by a variety of factors that are not taken into account in everyday clinical practice. The aim of this study was to evaluate the activity of platelet receptors in patients qualified for cardiac surgery, taking into account organ dysfunctions as well as pharmacological therapy applied in these patients.

## Material and method

**Fig. 1:**
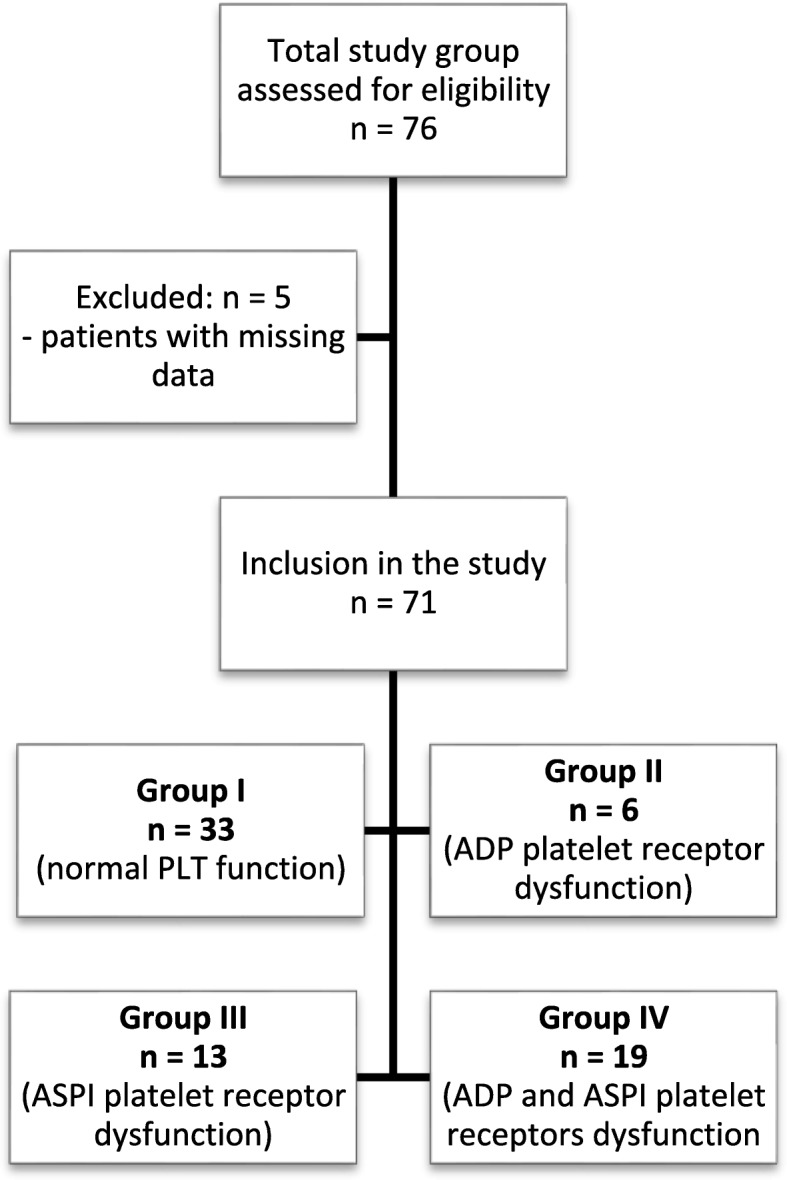
Study group selection

After approval by the Bioethical Commission at the Medical University in Wroclaw, the clinical data of patients operated on in the Department of Cardiac Surgery in 2015 were retrospectively retrieved. A total of *n* = 76 patients were assessed for eligibility. Of those, *n* = 5 were excluded through the lack of data, and *n* = 71 patients were enrolled in the study (Fig. 1). Two platelet receptors, most often blocked by antiplatelet agents, were evaluated using: ADP test determining ADP-induced platelet activation, sensitive to clopidogrel, prasurgel, and other ADP blockers, and ASPI test assessing acetylsalicylic acid-sensitive cyclooxygenase-dependent aggregation, NSAIDs and other platelet cyclooxygenase inhibitors. The results below the cut-off points: 570 AU x min for ADP test and 710 AU x min for ASPI test were considered to be the values with decreased activity of platelet receptors. The patients were divided into 4 groups (Table 1, Fig. 1). Group I (33/71 patients, without platelet dysfunctions), Group II (6/71 patients, ADP < 710 AU x min), Group III (13/71 patients, ASPI < 570 AU x min) and Group IV (19 / 71 patients, ADP < 710 AU x min and ASPI < 570 AU x min). Biochemical data defining the efficiency of the liver and kidneys, the list of preoperative drugs used and the requirement for postoperative transfusion throughout the study group were collected. Patients were routinely prepared for cardiac surgery. A small dose of ASPI receptor blockers was maintained up to the day before the procedure. ADP blocker therapy was discontinued 5 days prior to surgery as recommended by the ACC / AHA guidelines [25]. Platelet receptors dysfunction was assessed by the Multiplate® Analyzer that allows assessing the platelet function in a whole blood sample, based on the principle of multiple electrode aggregometry (MEA) [26, 27]. It is based on the fact that the platelets in the presence of added soluble agonist become active, exposes their receptors and promotes adhesion to the damaged vessel, in this case also to the artificial surface. MEA is measured by a special two sensory units composed of two electrodes each. The impedance and resistance between them are measured continuously in Ohms (Ω). When an agonist is added to the sample, the platelets begin to aggregate and settle on the electrodes, causing an increase in the impedance signal between them. The device registers this change in the form of a graph. The MEA expressed in Ohms is presented as an aggregation unit (AU). The measured area under the graph (AUC, 1 U = 10 AU x min) has a diagnostic value and is now a clinical parameter defining the receptor function [28].

**Table 1:**
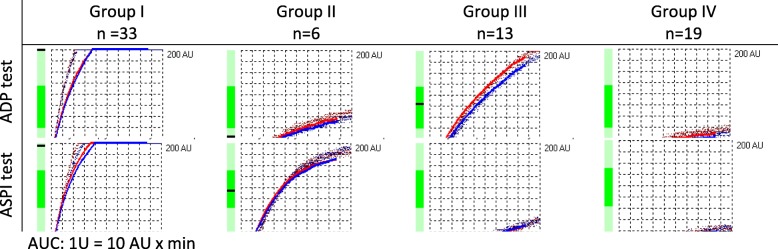
Division of the studied group depending on the activity of ADP and ASPI receptors

AUC: 1 U = 10 AU x min

### Statistical analysis

The obtained results were statistically analysed using Statistica 13.1 package (Statsoft, Poland).

Normality of the distribution of studied variables was verified with Shapiro-Wilk’s test. Due to the rejection of the hypothesis on the normality of the variables, the non-parametric tests (Mann-Whitney test, Spearman correlation, and Kruskal-Wallis ANOVA with post hoc test - multiple comparison test) were used in the study. The significance level of α = 0.05 was applied.

## Results

**Fig. 2:**
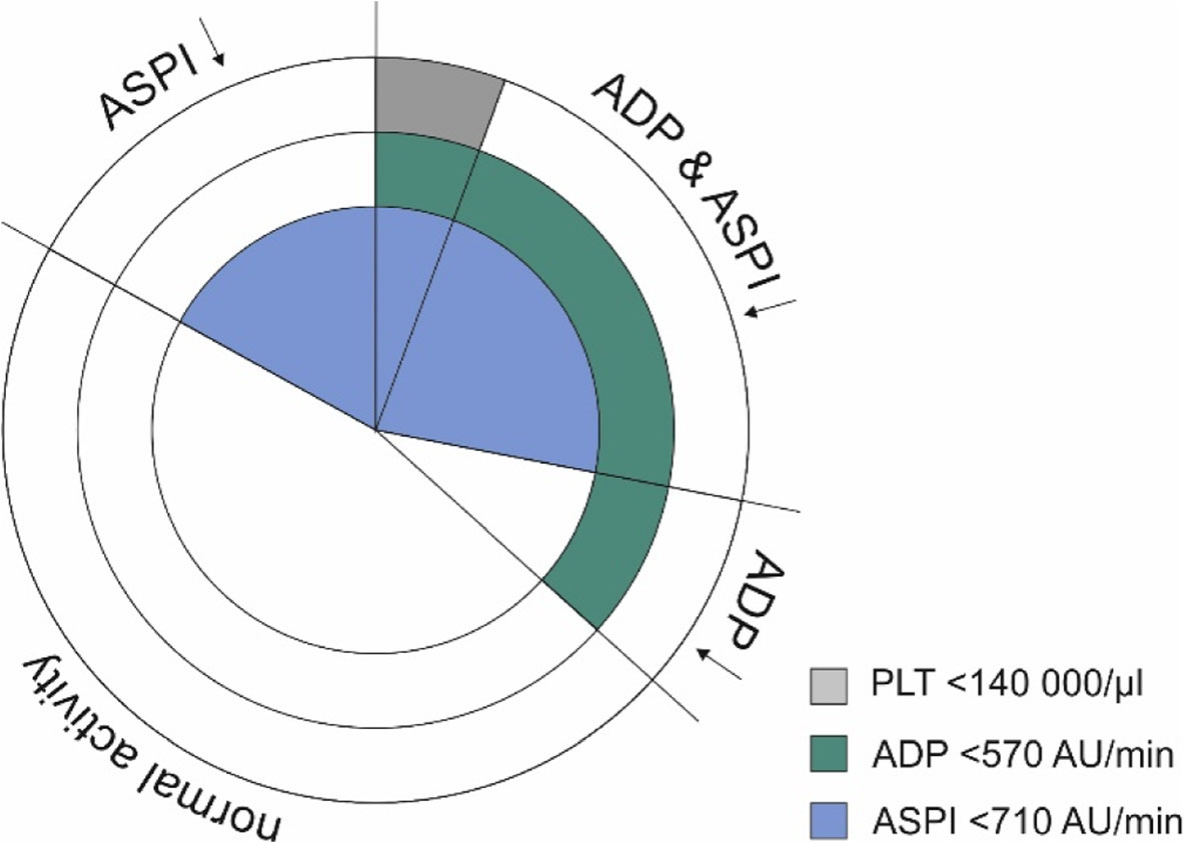
Distribution of ADP and ASPI receptor deficiency among the studied patients. Normal activity of both receptors was observed in 33 pts. (46.5%), decreased ADP in 6 pts. (8.5%), decreased ASPI in 12 pts. (17%), activity of both receptors was decreased in 20 pts. (28.2%)

**Fig. 3:**
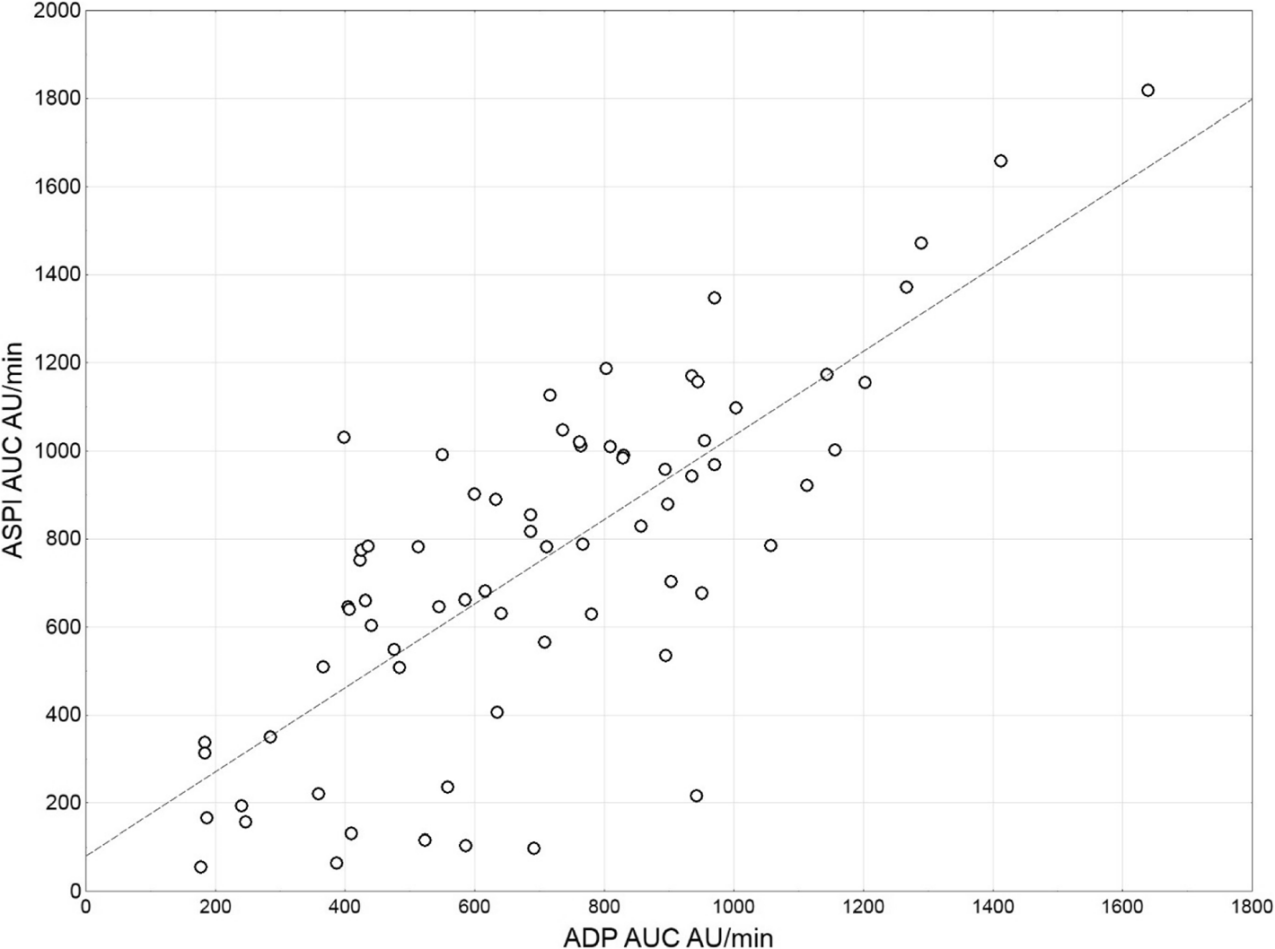
Dot plot of ADP and ASPI receptor activity showing a strong correlation between both receptors (rs = 0.72, *p* < 0.001)

**Fig. 4:**
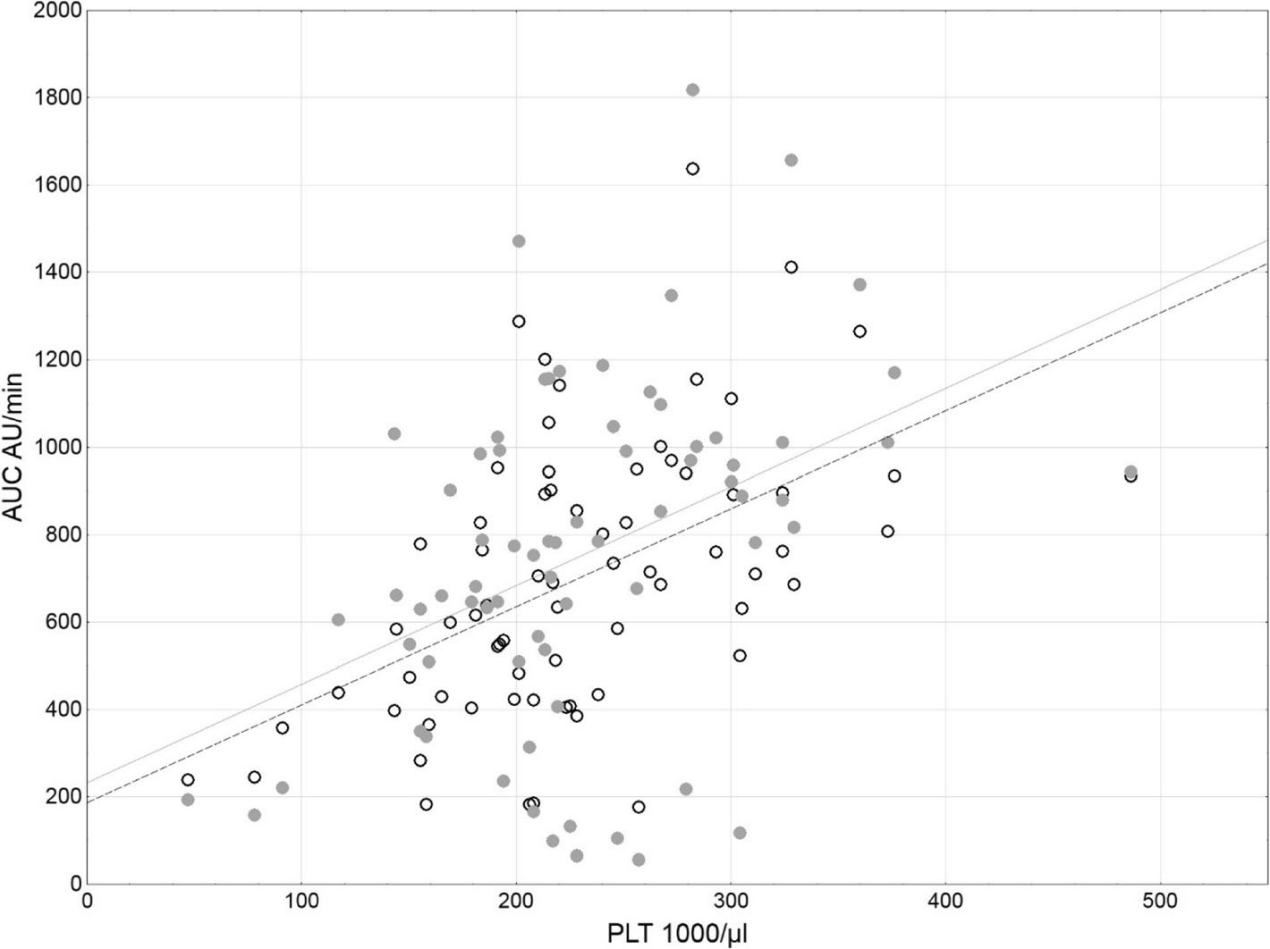
Dot plot of ADP (○, dashed line) and ASPI (●, gray line) receptor activity versus platelet count showing moderate correlations for both receptors (rs = 0.55, *p* < 0.001 and rs = 0.42, *p* < 0.001, respectively)

The study group included 41 males (57.7%) and 30 females (42.3%), mean age 66 years. All of the patients were subjected to cardiac surgery (*n* = 31 CABG alone; *n* = 40 other procedures). The averaged results of laboratory tests as well as ADP and ASPI tests are presented in Table 2. Most of the patients (94.4%) had platelet counts within the normal range. However, the platelet function was impaired in more than half of the studied patients (53.5%). 20 patients (28.2%) presented a combined dysfunction of ADP and ASPI receptors. 6 patients (8.5%) manifested only ADP receptor dysfunction and 12 (16.9%) only ASPI receptor dysfunction (Fig. 2). No relationship was found between the biochemical markers of the kidneys and liver and the function of the ADP and ASPI receptors (Table 3). ADP and ASPI activities were related (rs = 0.72, *p* < 0.001) and both associated with platelet count (rs = 0.55, *p* < 0.001 and rs = 0.42, *p* < 0.001, respectively) (Figs. 3, 4). The patients were divided into 4 groups according to ADP and ASPI receptor activity and their laboratory results were compared. Again, no difference was observed between the levels of biochemical markers of the kidneys and liver (Table 4). The platelet counts were the highest for the Group I – patients with normal receptor activity (275.2 ± 67.5), slightly decreased for patients with decreased ADP activity – Group II (199.7 ± 32.1, *p* = 0.006 vs Group I) or ASPI – Group III (210.3 ± 39.6, *p* = 0.003 vs Group I, *p* = 0.553 vs Group II), and the lowest in case of combined deficiency in receptor activity – Group IV (176.8 ± 61.6, *p* < 0.001 vs Group I, with no statistical significance when compared to single receptor deficiency). Platelet receptor activity was not related to the postoperative need for any type of transfusion. The need for RBC transfusion (26 pts.) was related only to lower hemoglobin or hematocrit levels (*p* = 0.025 and *p* = 0.044). The need for platelet transfusion was associated with higher INR and PT[%] (*p* = 0.024 and *p* = 0.033). No statistically significant correlation was found between preoperative pharmacotherapy and platelet receptors activity.

**Table 2:** The averaged results of laboratory tests

	Mean ± SD	median	% interpretation
Hemoglobin [g/dL]	13.0 ± 1.9	13.3	↓ 49.3%
Hematocrit [%]	38.9 ± 5.3	39.6	↓ 40.8%
Platelets [×10^3^/μL]	230.1 ± 73.1	218	↓ 5.6%
INR	1.06 ± 0.29	1.0	↑ 4.2%
PT [%]	97.2 ± 12.4	99.3	↑ 2.8%
Bilirubin [mg/dL]	0.81 ± 0.60	0.6	↑ 12.7%
ALAT	45.4 ± 90.7	29.0	↑ 22.5%
ASPAT	40.8 ± 53.3	26.0	↑ 29.6%
Urea [mg/dL]	49.6 ± 34.0	40.0	↑ 39.4%
Creatinine [mg/dL]	1.29 ± 1.12	1.07	↑ 28.2%
eGFR	63.3 ± 20.6	69.0	
ADP AUC, AU x min	702.9 ± 312.8	691	↓ 36.6%
ASPI AUC, AU x min	751.3 ± 390.1	782	↓ 45.1%

**Table 3:** Correlations between receptor activity and laboratory parameters (Spearman correlation coefficient and *p*-value)

	ADP		ADP/PLT		ASPI		ASPI/PLT	
	rs	p	rs	p	rs	p	rs	p
Hemoglobin [g/dL]	0.02	0.837	−0.14	0.231	0.07	0.538	0.01	0.943
Hematocrit [%]	0.04	0.757	−0.12	0.325	0.13	0.290	0.07	0.569
Platelets [×10^3^/μL]	0.55	< 0.001	−0.19	0.115	0.42	< 0.001	−0.23	0.049
INR	0.04	0.748	0.20	0.094	0.08	0.484	0.15	0.206
PT [%]	−0.04	0.747	−0.22	0.069	−0.06	0.641	−0.15	0.204
Bilirubin [mg/dL]	−0.08	0.505	0.03	0.816	0.05	0.705	0.10	0.400
ALAT	−0.03	0.785	−0.04	0.711	−0.00	0.983	0.02	0.894
ASPAT	−0.09	0.465	0.14	0.250	−0.06	0.635	0.12	0.325
Urea [mg/dL]	−0.01	0.910	0.10	0.400	0.03	0.796	0.16	0.183
Creatinine [mg/dL]	0.01	0.934	0.22	0.060	−0.07	0.553	0.11	0.374
eGFR	0.00	1.000	−0.21	0.086	0.18	0.126	−0.02	0.874

**Table 4:** The comparison of laboratory tests for the patients with various status of ADP and ASPI receptor activity

	Group I Mean ± SD Median (33)	Group II Mean ± SD Median (6)	Group III Mean ± SD Median (12)	Group IV Mean ± SD Median (20)	Kruskal Wallis ANOVA p	ADP normal or ↓	ASPI normal or ↓
						Mann Whitney p	Mann Whitney p
Hemoglobin [g/dL]	12.9 ± 1.8	13.4 ± 1.8	13.2 ± 1.2	12.9 ± 2.6	0.988	0.839	0.808
	13.3	13.2	13.3	13.4			
Hematocrit [%]	39.0 ± 4.7	40.0 ± 5.1	38.7 ± 3.2	38.7 ± 7.3	0.944	0.711	0.862
	40.0	39.0	38.9	39.9			
Platelets [×10^3^/μL]	275.2 ± 67.5	199.7 ± 32.1	210.3 ± 39.6	176.8 ± 61.6	< 0.001	< 0.001	< 0.001
	272	203.5	214.5	185.0			
INR	1.03 ± 0.10	1.03 ± 0.06	1.00 ± 0.07	1.16 ± 0.52	0.730	0.896	0.298
	1.02	1.03	1.00	1.00			
PT [%]	98.0 ± 7.8	97.7 ± 4.8	98.2 ± 8.5	95.0 ± 20.1	0.857	0.752	0.389
	98.2	97.6	100.3	100.0			
Bilirubin [mg/dL]	0.73 ± 0.40	0.95 ± 0.56	0.63 ± 0.26	1.01 ± 0.95	0.521	0.229	0.864
	0.06	0.70	0.55	0.65			
ALAT	41.8 ± 46.4	27.7 ± 10.7	27.3 ± 12.2	67.5 ± 160.5	0.849	0.620	0.972
	28.0	29.5	24.0	31.5			
ASPAT	35.4 ± 29.3	26.2 ± 6.2	26.9 ± 8.5	62.3 ± 90.8	0.370	0.283	0.481
	29.0	25.0	24.5	31.0			
Urea [mg/dL]	46.6 ± 26.0	54.3 ± 20.7	51.9 ± 56.7	51.8 ± 33.3	0.609	0.337	0.804
	40.0	50.0	39.0	40.0			
Creatinine [mg/dL]	1.37 ± 1.56	1.14 ± 0.49	1.27 ± 0.72	1.21 ± 0.36	0.651	0.587	0.239
	1.08	0.95	1.13	1.07			
eGFR	66.5 ± 22.5	67.5 ± 21.2	57.9 ± 15.6	60.1 ± 20.1	0.254	0.547	0.049
	71.0	75.5	59.5	61.0			
ADP AUC, AU x min	938 ± 237	457 ± 60	744 ± 143	364 ± 127	< 0.001	< 0.001	< 0.001
	897	430	699	395			
ADP/PLT	3.6 ± 1.2	2.3 ± 0.4	3.6 ± 0.7	2.3 ± 1.1	< 0.001	< 0.001	0.097
	3.5	2.2	3.4	2.3			
ASPI AUC, AU x min	1066 ± 243	853 ± 124	494 ± 229	356 ± 219	< 0.001	< 0.001	< 0.001
	1011	784	599	327			
ASPI/PLT	4.1 ± 1.2	4.5 ± 1.5	2.5 ± 1.4	2.3 ± 1.4	< 0.001	< 0.031	< 0.001
	3.8	3.8	2.7	2.4			

## Discussion

Platelet dysfunction is one of the causes of postoperative bleeding. Bleeding in cardiac surgery is of a remarkable importance factor in increased mortality. Ranucci et al. in a study, conducted on a group of 15,000 cardiac surgery patients, revealed that patients who suffered from massive bleeding were at increased risk of stroke, perioperative myocardial infarction, acute kidney injury, sepsis and significantly increased mortality from 2.6% up to 12.8% [29]. In our study, we performed *point-of-care* examinations before the procedure, defining the functioning of platelet receptors. The methods of diagnosis of these receptors can be divided into three categories: static tests, dynamic (non-activated) and tests of platelet responses to an agonist [30]. Based on the latter, the principle of the Multiplate® Analyzer is based. Its advantage is the evaluation of whole blood aggregometry (WBA) dysfunctions, i.e. in the presence of other cellular components, such as erythrocytes, which directly promote platelet aggregation, and monocytes that induce the formation of prostanoids [31]. This POC system is easy to use. Requires minimal technical effort and basic training. It can be performed outside a specialized laboratory - for example in an ICU or operating theatre. It is a useful tool for stratification of the risk of bleeding and the potential demand for blood products in cardiac surgery patients. This avoids unnecessary transfusions, as they not only save a life but also carry a significant risk of complications [32].

We confirmed a significantly impaired receptors activity in more than half of the studied patients (53.5%). No correlation was found between the weakening of receptors activity and the investigated harmful factors. Platelets in the aspect of the data presented on the physiology of coagulation are invariably an important and indispensable element protecting the correctness of hemostasis, and on the other hand, the complexity of this physiology makes them extremely sensitive to all harmful factors. Mentioned organ dysfunctions, biochemical disorders, and pharmacotherapy may negatively affect the thrombocytic cleavage potential. In addition, cardiac surgery performed with the use of a heart-lung machine and hypothermia may significantly increase their initial even a small dysfunction.

The affection of platelet receptors in the obtained results is significant. Decreasing the activity of the platelet receptors in the aspect of the planned cardiac surgery enables preparation for the treatment of the problem of postoperative bleeding by protecting the platelet concentrate. The total receptor blockade with the high risk of bleeding for ADP is < 310 AUC (31 U) and for ASPI < 300 AUC (30 U). In our study group, 16 patients (22.5%) experienced totally blocked platelet receptor, of which 5 patients (7%) both ADP and ASPI receptors at the same time. There are no established standards for preventive measures, especially based on the assessment of platelet receptors activity. Further preoperative observations study with the use of Multiplate® may contribute to the creation of procedures that will enable safer cardiac surgery in the extracorporeal circulation. An additional aspect of the described problem is antiplatelet therapy – more commonly used due to the increased incidence of cardiovascular diseases. Preoperative monitoring based on the POC principle is not widely used, however, it plays an increasingly important role, in particular in cardiac surgery departments. This study is important because an increasing number of patients requiring urgent intervention are treated with antiplatelet agents, including aspirin and P_2_Y_12_ ADP antagonists (i.e. clopidogrel, prasurgel, ticagrelor). As mentioned previously, early identification of these patients greatly facilitates the selection of an adequate therapeutic pathway. Pearse et al. in their work, showed that with the introduction of a bleeding management protocol, in which one of the components was the platelet receptors activity assessment, led to a decrease in the frequency of transfusions and the total number of blood products transfused as well as significantly reduced exploration surgery frere-quency, superficial chest and leg wound infections and length of the postoperative hospital stay [33].

In the conducted studies, it was shown that platelet receptors dysfunctions belong to one of the more frequent causes of coagulation system disorders. While the cause of some of them is possible to determine, there remains a large group of patients in whom the etiology of these dysfunctions we are unable to explain.

## Conclusions

Platelets are reactive morphotic elements of blood with a complex function and structure that play a key role in maintaining normal hemostasis. The function of platelet receptors impairs many internal pathologies and external factors, which in turn leads to a disruption of their participation in the coagulation process. Platelet dysfunction does not in every case lead to bleeding, but significantly increases its risk. The problem of intraoperative bleeding is extremely important as it affects the success of the surgery and its perioperative mortality. Preoperative POC diagnosis is an extremely useful tool that complements the clinical status of the patient. It is finding a wider and wider application in cardiac surgery departments, entering the standards of safe patient delivery through the procedure. The above study failed to prove the relationship between platelet receptors disorders and mild to moderate liver and kidney damage as well as preoperative pharmacological therapy applied in patients qualified for cardiac surgery. However, platelet receptors dysfunction has been shown to be associated with a decreased number of platelets. The pathophysiology of platelet disorders is still not fully understood and requires further research and observation.

## Abbreviations

ACC: American College of Cardiology
ADP: Adenosine diphosphate
AHA: American Heart Association
ALAT: Alanine transaminase
ANOVA: Analysis of variance
APTT: Activated partial thromboplastin time
ASA: Acetylsalicylic acid
ASPAT: Aspartate transaminase
AU: Aggregation units
AUC: Area under the curve
cAMP: Cyclic adenosine monophosphate
DIC: Disseminated intravascular coagulation
eGFR: Estimated glomerular filtration rate
ESRD: End-stage renal disease
GP: Glycoprotein
HIT: Heparin-induced trombocytopenia
HUS: Hemolytic-uremic syndrome
ICU: Intensive care unit
INR: International normalized ratio
MEA: Multiple electrode aggregometry
NSAIDs: Nonsteroidal anti-inflammatory drugs
POC: Point-of-care
PT: Prothrombin time
RBC: Red blood cell
vWF: von Willebrand factor
WBA: Whole blood aggregometry
